# A Stratified Transcriptomics Analysis of Polygenic Fat and Lean Mouse Adipose Tissues Identifies Novel Candidate Obesity Genes

**DOI:** 10.1371/journal.pone.0023944

**Published:** 2011-09-07

**Authors:** Nicholas M. Morton, Yvonne B. Nelson, Zoi Michailidou, Emma M. Di Rollo, Lynne Ramage, Patrick W. F. Hadoke, Jonathan R. Seckl, Lutz Bunger, Simon Horvat, Christopher J. Kenyon, Donald R. Dunbar

**Affiliations:** 1 Molecular Metabolism Group, BHF/University Centre for Cardiovascular Science, University of Edinburgh, Queen's Medical Research Institute, Edinburgh, United Kingdom; 2 Tissue Remodelling and Regeneration Group, Centre for Inflammation Research, Queen's Medical Research Institute, Edinburgh, United Kingdom; 3 Endocrinology Unit, BHF/University Centre for Cardiovascular Science, University of Edinburgh, Queen's Medical Research Institute, Edinburgh, United Kingdom; 4 Animal Breeding and Development Team, Sustainable Livestock Systems, SAC, Bush Estate, Penicuik, United Kingdom; 5 Animal Science Department, Biotechnical Faculty, University of Ljubljana, and National Institute of Chemistry, Hajdrihova, Ljubljana, Slovenia; 6 Bioinformatics Core, BHF/University Centre for Cardiovascular Science, University of Edinburgh, Queen's Medical Research Institute, Edinburgh, United Kingdom; University of Tor Vergata, Italy

## Abstract

**Background:**

Obesity and metabolic syndrome results from a complex interaction between genetic and environmental factors. In addition to brain-regulated processes, recent genome wide association studies have indicated that genes highly expressed in adipose tissue affect the distribution and function of fat and thus contribute to obesity. Using a stratified transcriptome gene enrichment approach we attempted to identify adipose tissue-specific obesity genes in the unique polygenic Fat (F) mouse strain generated by selective breeding over 60 generations for divergent adiposity from a comparator Lean (L) strain.

**Results:**

To enrich for adipose tissue obesity genes a ‘snap-shot’ pooled-sample transcriptome comparison of key fat depots and non adipose tissues (muscle, liver, kidney) was performed. Known obesity quantitative trait loci (QTL) information for the model allowed us to further filter genes for increased likelihood of being causal or secondary for obesity. This successfully identified several genes previously linked to obesity (*C1qr1*, and *Np3r*) as positional QTL candidate genes elevated specifically in F line adipose tissue. A number of novel obesity candidate genes were also identified (*Thbs1*, *Ppp1r3d*, *Tmepai*, *Trp53inp2*, *Ttc7b*, *Tuba1a*, *Fgf13*, *Fmr*) that have inferred roles in fat cell function. Quantitative microarray analysis was then applied to the most phenotypically divergent adipose depot after exaggerating F and L strain differences with chronic high fat feeding which revealed a distinct gene expression profile of line, fat depot and diet-responsive inflammatory, angiogenic and metabolic pathways. Selected candidate genes *Npr3* and *Thbs1*, as well as *Gys2*, a non-QTL gene that otherwise passed our enrichment criteria were characterised, revealing novel functional effects consistent with a contribution to obesity.

**Conclusions:**

A focussed candidate gene enrichment strategy in the unique F and L model has identified novel adipose tissue-enriched genes contributing to obesity.

## Introduction

Obesity and its co-associated metabolic diseases result from a complex interaction between environmental and genetic factors [1]–[3]. The polygenic Fat (F) and Lean (L) mouse models were selectively bred for divergent body fat mass [4] and thus model complex polygenic human obesity. Four major obesity QTLs in the F line were initially described in an F_2_ cross of the out-bred F and L mouse lines [5]. Notably, obesity in the F line is independent of leptin, the leptin receptor and other characterised single gene obesity mutations relating to central control of appetite or energy balance [5], [6]. Indeed, F mice have a lower caloric intake than L mice [6], [7]. Since the divergent adiposity of the model was selected using fat pad mass rather than food intake, and because several studies, including recent meta-genome wide association, link lipid metabolism and intrinsic molecular pathways within the adipose tissue to fat mass regulation [8]–[12] we reasoned that the F and L adipose tissue would be a rich resource for identifying obesity-susceptibility genes.

We describe a stratified microarray analysis of gene expression using first a qualitative, comparative ‘snap-shot’ pooled transcriptomic approach across several adipose tissue depots and non-adipose metabolic tissues to broadly identify adipose tissue-specific gene expression differences between F and L lines. We used previously defined QTL information [5] to enrich for genes with an increased causal likelihood. This was followed by quantitative gene chip validation in subcutaneous fat, where the largest divergence in fat mass in response to chronic high fat (HF) feeding was observed [13]. Having defined adipose-tissue specific pathways and positional candidate obesity genes we performed real time PCR (RT-PCR) validation of key affected pathways. We then functionally linked two of the QTL-associated candidate obesity genes – and one non-QTL associated gene that had an expression profile parallel to that of the candidate genes – to fat cell function.

## Results and Discussion

### Insights into adipose tissue and depot-specificity of gene expression: Snap-shot results

**Figure 1:**
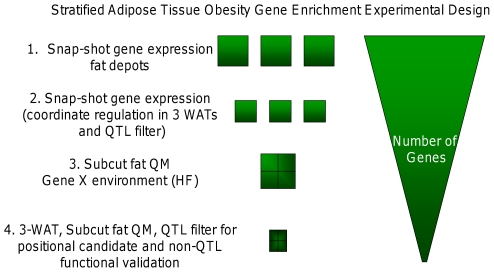
A stratified transcriptomics approach for adipose tissue-specific gene enrichment. Schematic of the experimental design used to enrich for adipose tissue- genes candidate obesity genes. Several successive filters were applied that decreased the numbers of genes fulfilling the inclusion criteria (right hand panel). From top to bottom; 1. differential gene expression (>1.5-fold) between the lines across all adipose tissues was analysed with a ‘snap-shot’ pooled transcriptomics approach. 2. Adipose selective gene expression was then considered by including only genes that were co-ordinately differentially expressed between the lines in the 3 white fat depots. 3. Increased stringency (≥2-fold difference) and filtering with known obesity QTL boundaries [5] were used to select for an increased likelihood that the differentially expressed genes were causal for divergent adiposity. 4. A gene-environment interaction was modelled by identifying genes that were differentially expressed in a quantitative microarray (QM) analysis of the subcutaneous adipose tissue after high fat feeding. Some genes fulfilling all or most of these criteria were validated.

We applied a 4 step filtering strategy to narrow our search for key adipose-specific obesity-associated genes (Figure 1) as described in Methods and Materials.

The number of genes that were differentially expressed between the lines across any of the three fat depots (3WATs) was markedly reduced after the first filter was applied to enrich for genes co-ordinately regulated in 3WATs (Table 1, Figure 1). Notably, in terms of overall adiposity (3 WATs) there were 10 times as many genes elevated in F (102) than L mice (9). This may reflect that causal gene variants raising the upper set-point of body fat mass are not as strongly proscribed, in the absence of predation, in contrast to genes conferring a potentially threatening low fat mass [14]. Despite EPI fat being the sole selection criteria for the first 20 generations of the breeding [4] there were fewer differences in gene expression in the EPI of F mice (380 versus SC 426 and MES 450). This may reflect the induction of secondary inflammatory, metabolic, angiogenic and tissue remodelling programmes that are more pronounced in the F line SC and MES adipose tissues (see below). This is also consistent with the SC being the most divergently HF responsive tissue [13] and with visceral (MES) fat being highly vascularised and immune cell-rich. In contrast, twice as many differentially expressed genes were elevated in the EPI of L mice (484 versus SC 262 and MES 201) which may reflect an EPI depot-selective process that is relatively quiescent in other L fat depots.

**Table 1:** Gene numbers associated with differential expression in adipose and grouped for QTL position.

number of total/known* genes matching criteria					
FAT genes	Genomewide	Fob1 (%)	Fob2 (%)	Fob3(%)	Fob4(%)
↑ SC	426/355	22/21 (6)	9/82)	11/11(3)	11/10(3)
↑ EPI	380/319	20/17 (5)	12/10(3)	9/8(3)	12/12(4)
↑ MES	450/378	28/25 (7)	9/9(2)	13/12(3)	8/7(2)
**↑3 WATsF**	102/89	7/7(8)	3/3(3)	1/1(1)	1/1(1)
**LEAN genes**					
↑ SC	262/228	8/7(3)	4/3(1)	7/6(3)	4/4(2)
↑ EPI	484/402	12/12(3)	18/13(3)	16/12(3)	19/16(4)
↑ MES	201/177	4/4(2)	6/4(2)	6/5(3)	7/6(3)
**↑3 WATsL**	9/9	0/0( )	0/0( )	1/1(11)	0/0( )

Number of genes expressed >1.5-fold between the F and L lines in 3 distinct white adipose depots when fold-changes in muscle, liver and kidney were set to be between <1.5-fold different between F and L lines. The number of genes co-ordinately regulated in all 3 white fat depots (3 WAT) reduces the number of genes to those with a broad functional role (not depot-specific) in fat cell function (see text). FAT genes refer to genes with expression higher in F-line adipose tissue and LEAN genes refer to genes with expression higher in L-line adipose tissue. ↑ = increased expression, ↓ = decreased expression. The number after the/oblique in blue refers identified Entrez ID genes. The number in parenthesis indicates the number of genes within a QTL [5] as a percentage of the whole (genome wide column).

The genes found by the relaxed 3WAT criteria were exported to Webgestalt and screened against the Kyoto Encyclopaedia of Genes and Genomes (KEGG) and Gene Ontology database to look for enrichment of functional pathways that were over-represented in the F adipose tissue (Table 2). Database for Annotation, Visualization and Integrated Discovery (DAVID) was also used for pathway analysis with similar findings (data not shown).

**Table 2:** Gene set enrichment analyses of ‘snapshot’ and quantitative array data.

Database	Category	Observed number	Expected Number	P-Value
**Upregulated 3WAT (368 genes)**				
GOBP	fat cell differentiation	8	1	0.002
GOBP	cell adhesion	22	7	0.002
GOBP	cell death	26	11	0.007
GOBP	cell-matrix adhesion	6	1	0.007
KEGG	ECM-receptor interaction	10	1	2e-6
KEGG	PPAR signaling pathway	6	1	0.005
**Downregulated 3WAT (113 genes)**				
GOBP	antigen processing and presentation of peptide antigen	5	<1	0.0001
GOBP	immune response	11	2	0.0005
KEGG	Antigen processing and presentation	4	<1	0.0008
**Upregulated 3WAT not regulated in liver, muscle, kidney (165 genes)**				
GOBP	cell adhesion	13	4	0.05
KEGG	ECM-receptor interaction	7	<1	1e-5
**Downregulated 3WAT not regulated in liver, muscle, kidney (14 genes)**				

No significant enrichment				
Database	Category	Observed number	Expected Number	P-Value
**Upregulated FFvFC (350 genes) in the quantitative array**				
GOBP	Lipid metabolic process	40	12	2e-8
GOBP	Cell adhesion	31	10	4e-6
KEGG	PPAR signaling pathway	9	1	2e-5
KEGG	Glycerophospholipid metabolism	8	1	0.0001
**Downregulated FFvFC (39 genes) in the quantitative array**				
GOBP	Immunoglobulin mediated immune response	3	<1	0.048
KEGG	Hematopoietic cell lineage	4	<1	6e-5

In the snapshot data, enriched categories are shown for genes differentially expressed in all three white adipose tissues (3WAT; >2-fold; >100AU in higher expressing strain) between fat and lean mice. In the quantitative array data, enriched categories are shown for genes differentially expressed between Fat mice on a high fat diet and Fat mice on a control diet (>2-fold; p-value<0.05, >100AU in higher condition). GOBP = Gene Ontology Biological Process; KEGG = Kyoto Encyclopedia of Genes and Genomes pathway database. Data analysed in Webgestalt, similar results using DAVID and Metacore (not shown).

Table 3 shows the list of genes identified from more stringent criteria (≥2-fold difference between the lines in 3WATs) that were additionally positioned within major QTL boundaries [5]. This further reduced the chance of selecting functionally neutral or secondary genes. Three of these genes (*C1qr1*, *Np3r and Thbs1*) have been previously linked to obesity or adipose tissue function (Table 3). Several novel adipose-tissue specific mechanisms potentially contributing to fat mass accumulation and/or its associated metabolic consequences are inferred from functions ascribed to the other genes or closely-related functionally characterised genes or disease processes (Table 3).

**Table 3:** Genes with increased causal likelihood as indicated by a stringent stratified criteria.

*Up in F line adipose tissue*			
Gene (Mean fold-expression difference across 3 WATs)	*function(s)?(gene functions abridged from iHOP: * http://www.ihop-net.org *). Some functions are inferred from associated phenotypes and are denoted by* (?)	*Linked with obesity? (mean expression intensity across 3WAT ‘snap-shot’ arbitrary units)*	*[Refs]*
***Fob1***			
*Thbs1* (3.4)	Angiogenesis, platelet and macrophage function, fibrogenesis	Yes(∼402)	[39]–[42]
*C1qr1* (2)	Mac/NK cell phagocytosis, lectin binding	Yes (∼300a.u.)	[19]
*Ppp1r3d* (2.5)	Regulatory subunit of phosphatase1, glycogen synthesis. There are ∼16 *Ppp1rs*	No (∼100a.u.)	[61: *Ppp1r3a*]
*Tmepai* (4)	Transmembrane egf/androgen response, cell cycle. Obesity and androgen signalling (?)	No(∼390a.u.)	[62]
*Trp53inp2* (3)	embryogenesis: neural tissue, p53-inducible	No(∼3060a.u.)	[63]
***Fob2***			
*Ttc7b* (2.5)	Unknown. Related WD40/tetracopeptide involved in lipid storage(?)	No(∼560a.u.)	[64]
*Tuba1a* (2)	cytoskeleton/structural/transportlipid droplet-associated	No(∼4265a.u.)	[65]
***Fob3***			
*Npr3* (7)	Natriuretic peptide clearance receptor	Yes(∼560a.u.)	[36]–[37]
***Fob4***			
*Fgf13* (8)	morphogenesis, tissue repair. Other *Fgfs* have a role in adipose tissue and metabolism?	No (∼425a.u.)	[66]–[67]
*Fmr1*(4.5)	cell signalling, RNA binding, neuronal: Related to Prader-Willi –like obesity syndrome in fragile X syndrome?	No(∼1485a.u.)	[68]–[69]

A stringent search, based on QTL boundaries (Horvat et al., 2000, ref 5) produces a limited set of genes with ≥*2fold* increased expression levels in all *3 WATs* of F when the difference in gene expression is set between −2 and +2 in muscle, liver and kidney. Only genes with a higher mean intensity of 100 a.u. in the 3 WATS were selected (more likely to be of functional relevance in adipose tissue).

### ‘Snap-shot’ filters for non-adipose specific divergently expressed genes

The base population for the F and L strains was created by crossing two inbred and one out-bred strain [4]. Line-specific differences in gene expression that are potentially unrelated to adiposity (neutral) may therefore co-segregate with QTLs in an F_2_ cross or are secondary due to vast differences in final phenotype/pathology between the lines. Differential expression due to sequence divergence between F and L genomes may also result in false-positive expression differences due to altered microarray hybridisation efficiency. To help address this, the ‘snap-shot’ analysis was used to exclude genes that were differentially expressed across all tissues. Thus, gene expression was compared between 3 representative adipose depots versus muscle, liver and kidney using tissues pooled from 3 mice from each line as described in Materials and Methods. Examples of divergently expressed, but non-adipose-specific, genes (*Depdc6* [15], [16], *Gsn*, Table 4) were not further pursued in the present study, although we do not discount their potential functional relevance to the phenotype of F or L lines [17].

**Table 4:** Exclusion of non-adipose-specific (all-tissue) divergently expressed genes.

	*Depdc6 F-line gene expression*					
	SC	EPI	MES	LIV	MUSCLE	KIDNEY
Fat	1392	1506	947	791	888	263
Lean	10	11	14	18	12	12
**fold-change**	**138**	**133**	**68**	**44**	**75**	**23**

	*Gsn L-line gene expression*					
	SC	EPI	MES	LIV	MUSCLE	KIDNEY
Fat	10	6	6	9	9	6
Lean	8455	10420	4408	51	444	227
**fold-change**	**−864**	**−1646**	**−613**	**−6**	**−444**	**−41**

Numbers represent absolute chip expression intensity (AU) values from pooled transcriptome Genechip 2.0 array of subcutaneous (SC) epididymal (EPI) mesenteric (MES) and liver, muscle and kidney. Note, due to the mean expression intensity being <100 AU, *Depdc6* and *Gsn* are effectively switched on in the Fat and Lean line tissues, respectively. Positive fold-change numbers indicate elevated in F line. Negative Ratio numbers indicate elevated in L line.

### Modelling a gene-by-diet interaction: further insight from quantitative microarray

**Figure 2:**
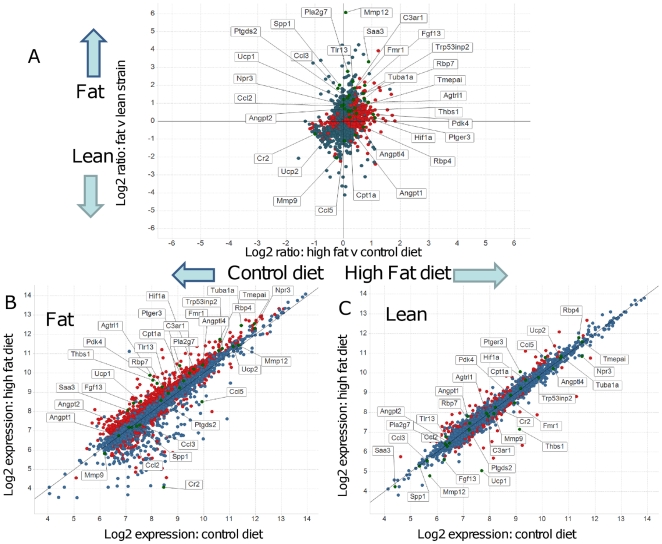
Differential gene expression in subcutaneous adipose tissues of Fat and Lean mice exposed to control or high fat diets. **A.** Log (base 2) ratios of gene expression intensities in fat and lean mice on the high fat and control diets. The y-axis shows the comparison of fat and lean strains regardless of diet. The x-axis compares high fat and control diets regardless of strain. Red spots represent genes that are significantly differentially expressed in Fat mice on high fat diet compared to control diet. Several genes of interest with higher expression in Fat mice and on a high fat diet are marked and labelled. **B.** and **C.** Log (base 2) gene expression intensities in (B) Fat mice and (C) Lean mice on the high fat and control diets. For each strain, the y-axis shows the log2 expression in high fat fed mice. The x-axis is for control diet. Red spots represent genes that are significantly differentially expressed in high fat diet compared to control diet for each strain. Several genes of interest discussed in the text are marked and labelled on graphs for both strains. Genes expressed below the arbitrary threshold (100) throughout the experiment were removed for clarity.

To model a gene-by-diet interaction and potentially amplify responses of obesity-susceptibility genes we performed quantitative analysis (n = 4 per group) of the transcriptome in the SC fat depot. SC fat was chosen as it showed the greatest phenotypic divergence (fat mass) in response [13] to the obesigenic stimulus of chronic HF feeding (Figure 2, Table 5). Notably, with the statistical power of the quantitative microarray, far fewer genes were differentially expressed in subcutaneous fat between the lines under control conditions (Table 1) demonstrating the importance of the quantitative approach for reducing false positives. The baseline gene expression differences were clearly amplified by diet (Table 5). As with basal differences between the lines, the F line had a more marked gene expression response to the diet than the L line (Figure 2, Table 5). The top functional pathways affected in F line obesity are listed in Table 2 and reflect an accentuation of changes in angiogenesis, cell migration, UDP-glycosylation, prostaglandin synthesis, triglyceride and glycogen synthesis, collagen formation, ER and stress fibre pathways and membrane peptidases.

**Table 5:** Quantitative gene-chip analysis results showing the numbers of genes differentially expressed >2 fold with an adjusted p = value<0.05, and where the mean expression is >100 a.u. intensity for the higher group.

*number of upregulated genes matching criteria*					
	Genomewide	Fob1 (%)	Fob2 (%)	Fob3(%)	Fob4(%)
FC vs LC	98	3(3)	0(0)	0(0)	5(5)
FF vs LF	254	13(5)	4(2)	3(1)	12(4)
FF vs FC	95	4(4)	0(0)	2(2)	5(5)
LF vs LC	27	0(0)	0(0)	2(7)	3 (1)

*number of downregulated genes matching criteria*					
	Genomewide	Fob1 (%)	Fob2 (%)	Fob3(%)	Fob4(%)
FC vs LC	87	7(8)	2(2)	1(1)	2(2)
FF vs LF	225	6(3)	2(1)	5(2)	7(3)
FF vs FC	21	0(0)	0(0)	1(5)	1(5)
LF vs LC	16	1(6)	0(0)	2(13)	0(0)

Upregulated (A) refers to genes that are higher in the first group in the row compared to (vs) the second group in the row. Downregulated (B) refers to genes that are lower in the first group in the row compared to (vs) the second group in the row. For example upregulated in FC vs LC means higher expression in **F**at line **C**ontrol fed than in (vs) **L**ean line **C**ontrol fed. Only diet based (high fat v control diet) and strain based (Fat v Lean) comparisons are shown. All results are from the subcutaneous fat pad that showed the most divergent response to high fat (HF) feeding. FC; Fat line control diet, FF; Fat line HF diet, LC; Lean line control diet, LF; Lean line HF diet. The left column shows the total number of differentially expressed genes (Genome wide) and then those that lie within the 95% confidence interval of the major QTLs Fobs1-4 [5]. This number is also expressed as a percentage of the Genome wide number (%) in parentheses.

### Validation of key changes in gene expression within the context adipose tissue dysfunction in obesity

**Figure 3:**
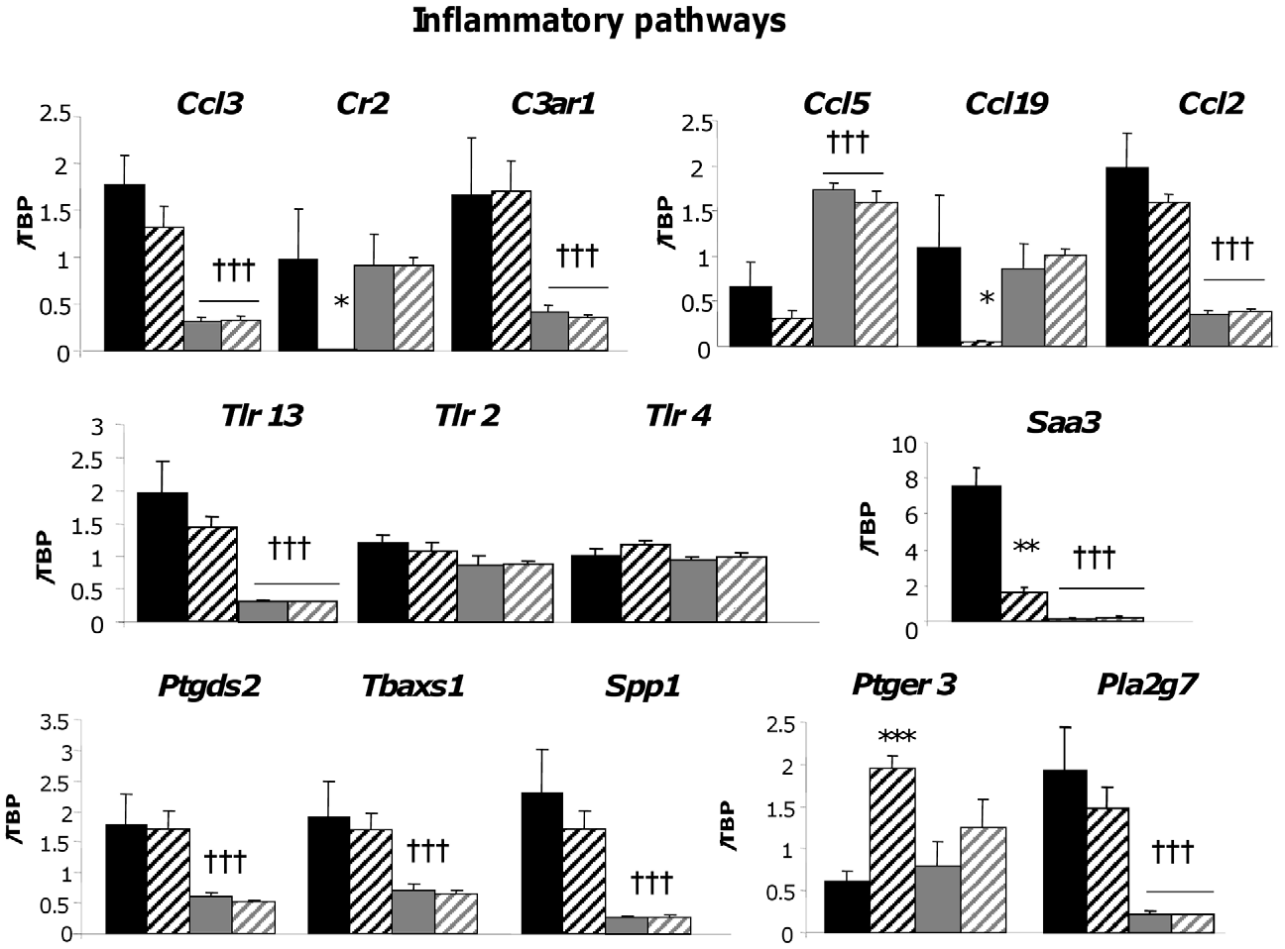
RT-PCR validation of inflammatory genes differentially expressed in the subcutaneous adipose tissue of Fat and Lean mice. Fat and Lean mice were fed control (FC; black bars, LC; grey bars) or high fat diet (FF; black hatched bars, LF; grey hatched bars) for 18 weeks. RNA was extracted and the gene specified above the graphs was measured by RT-PCR. Target gene expression was corrected to the expression level of the housekeeping gene tata-binding protein (TBP) and is expressed arbitrarily as an adjusted ratio. * = P<0.05, ** = P<0.01 significant effect of diet within a line. ††† = P<0.001 significant difference between lines.

**Figure 4:**
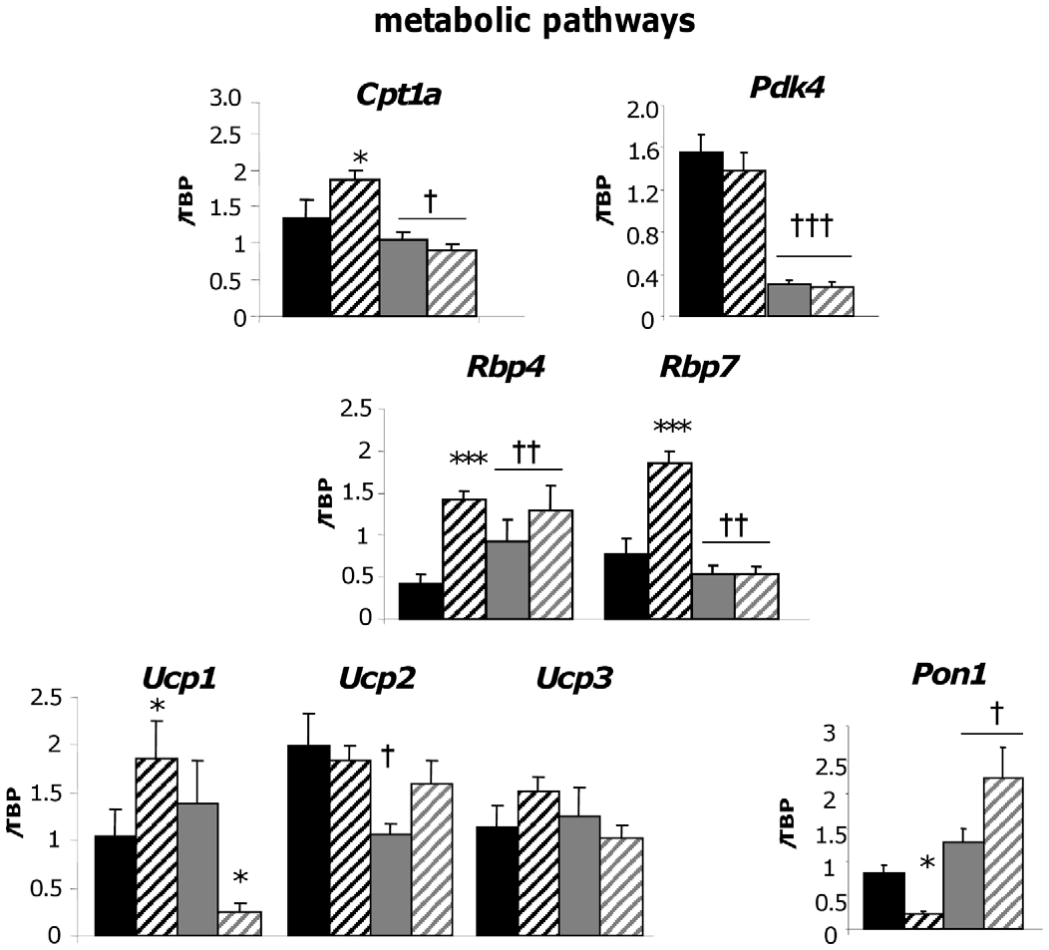
RT-PCR validation of metabolic and anti-oxidant genes differentially expressed in the subcutaneous adipose tissue of Fat and Lean mice. Fat and Lean mice were fed control (FC; black bars, LC; grey bars) or high fat diet (FF; black hatched bars, LF; grey hatched bars) for 18 weeks. RNA was extracted and the gene specified above the graphs was measured by RT-PCR. Target gene expression was corrected to the expression level of the housekeeping gene tata-binding protein (TBP) and is expressed arbitrarily as an adjusted ratio. * = P<0.05, *** = P<0.001 significant effect of diet within a line. † = P<0.05, †† = P<0.01, ††† = P<0.001 significant difference between lines.

**Figure 5:**
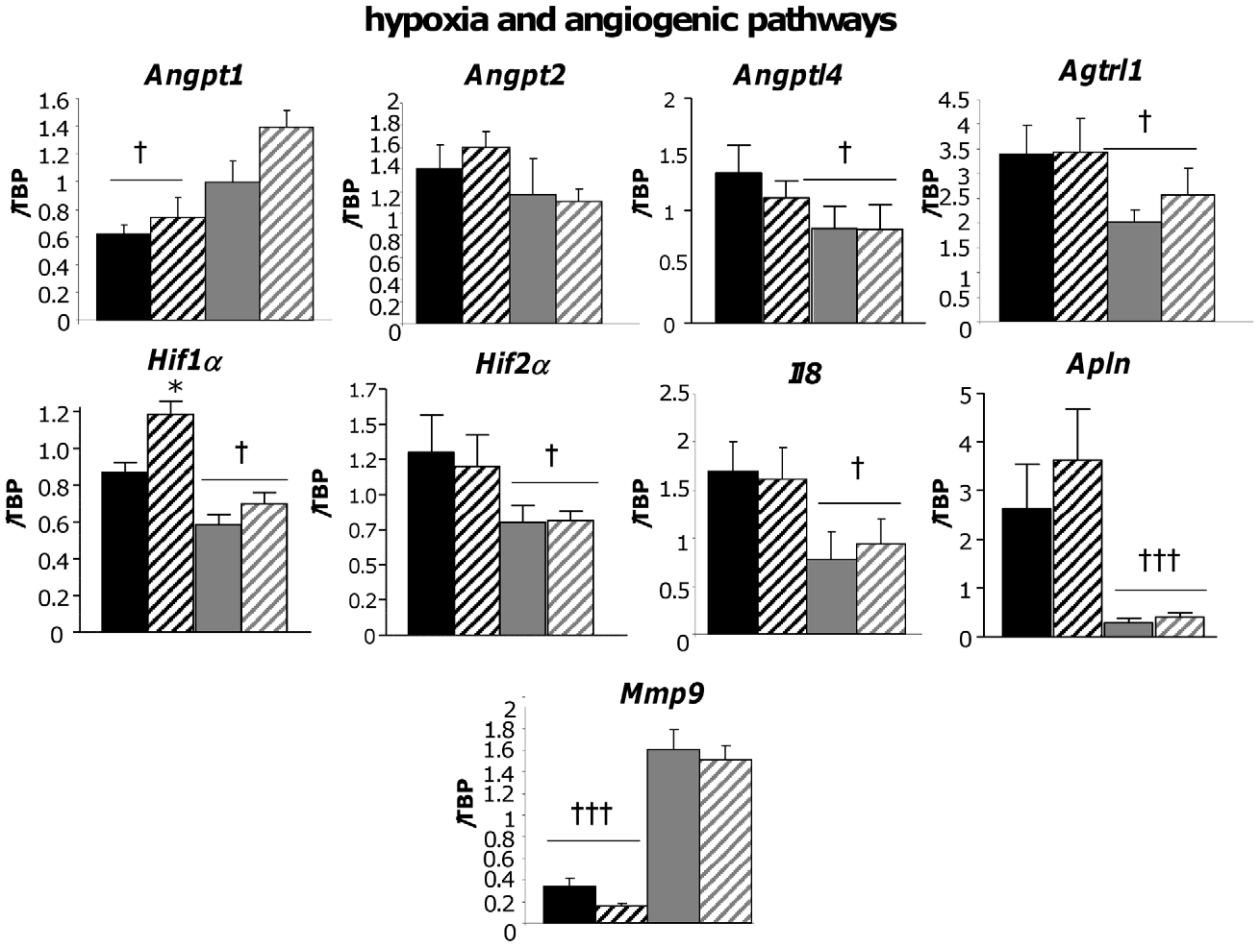
RT-PCR validation of angiomodulatory genes differentially expressed in the subcutaneous adipose tissue of Fat and Lean mice. Fat and Lean mice were fed control (FC; black bars, LC; grey bars) or high fat diet (FF; black hatched bars, LF; grey hatched bars) for 18 weeks. RNA was extracted and the gene specified above the graphs was measured by RT-PCR. Target gene expression was corrected to the expression level of the housekeeping gene tata-binding protein (TBP) and is expressed arbitrarily as an adjusted ratio. * = P<0.05 significant effect of diet within a line. † = P<0.05, ††† = P<0.001 significant difference between lines.

Line differences in key genes involved in the inflammatory (Figure 3), metabolic and anti-oxidant (Figure 4) and hypoxia/angiogenic (Figure 5) pathways were validated by RT-PCR in a larger cohort (n = 8–11). This gave insight into line and diet-specific regulation of key genes contributing to obesity and the associated functional changes in the adipose tissue in the most phenotypically divergent fat depot.

### Line and diet effects on inflammatory genes

Our analyses revealed a clear line divergence of inflammatory gene expression with distinct and sometimes unexpected HF diet responses. Most of the genes in this pathway were not obesity QTL-associated and hence represent secondary responses (Figure 3 and microarrays). Many inflammatory genes that were more highly expressed in F adipose tissue have been linked with obesity and/or insulin resistance, (*Ccl3*/MIP1ά [18] *C3ar1* [19], *Ccl2*/MCP1 [20], *spp1*/osteopontin [21]). Others appear to link obesity and inflammation with vascular function and haemostasis (*Ptgds2*, *Tbaxs1*, and *Pla2g7* [note *Pla2g16* has been linked with obesity ref. 10], *Tlr13*). Surprisingly, *Tlr13* (mapping to *Fob4*) expression, but not the *Tlr2* or *Tlr4* genes encoding the canonical proinflammatory bacterial lipotoxin receptors (e.g. lipopolysaccharide) recently shown to mediate adipocyte pro-inflammatory and insulin resistance effects of free fatty acids [22], was higher in F adipose tissue. *Tlr13* is most closely related to *Tlr3* that modulates innate immune responses [23] and, unlike TLRs1-9 [24], has not been linked to adipose tissue biology. Of note, *Tlr13* mRNA expression is higher in extreme obesity/diabetes mouse strains (Supplementary Figure S1). Notably, some immunomodulatory genes were expressed more highly in L adipose tissue indicating an alternate inflammatory response (*Ccl5*/RANTES [25]).

Striking diet-sensitive gene expression patterns were also observed. For example, mRNA levels for *Cr2* (CD21) and *Ccl19* (MIP3β) that link innate and adaptive immunity and dendritic cell migration [26] were markedly suppressed by HF diet in the F line. In contrast, HF diet induced proinflammatory *Ptger3* expression in F adipose tissue but suppressed obesity-associated [27] serum proinflammatory *Saa3* expression. The divergence in discrete immune responses may help identify pathological versus reparative processes and informs the simplistic view of a general increase in inflammatory gene expression in adipose tissue in obesity.

Overall we saw evidence of a selectively activated proinflammatory adipose environment which is modulated by HF diet in F adipose tissue but also an anti-inflammatory profile in adipose tissue of obesity-resistant L mice with very little response to HF diet.

### Line and diet effects on metabolic genes

We found a major divergence in gene expression for lipid and carbohydrate metabolism (Figure 4 and microarray). Genes involved in fat oxidation (*Cpt1*) and energy dissipation (*Ucp2*) were elevated in the F adipose tissue and this was induced further with HF diet. Of note, uncoupling protein 1 (*Ucp1*) – a major mitochondrial protein linked to energy expenditure and obesity-resistance [28] – was down regulated with HF in L adipose tissue, indicating that healthy leanness was not due to increased thermogenic drive in WAT. However, increased mRNA levels for the lipolytic β3-adrenergic receptor (*Adrb3*) (quantitative microarray data) suggests increased release of adipocyte fatty acids, consistent with reduced adiposity which, in combination with increased skeletal muscle fat oxidation in the L line [29], is consistent with healthy leanness. The secreted retinol binding protein4 (*Rbp4*) – associated with obesity and insulin resistance [30] – was unexpectedly higher in L adipose tissue (Figure 4), although it was HF-inducible in the F line. *Rbp4* may therefore have a context dependent relationship with insulin sensitivity. Intriguingly, intracellular *Rbp7* was elevated in F adipose tissue and was further induced with HF. *Rbp7* thus represents a novel obesity-induced gene. Altered retinol metabolism appears to be a feature of adipose tissue in obesity [9]. Lower mRNA levels of the HDL-associated anti-oxidant factor *Pon1* [31] were found in F adipose tissue. HF diet suppressed *Pon1* further in F adipose tissue but induced *Pon1* expression in the L adipose tissue (Figure 4) suggesting an enhanced anti-oxidant response in L adipose associates with their improved metabolic profile.

Consistent with impaired carbohydrate metabolism, pyruvate dehydrogenase kinase 4 (*Pdk4*) mRNA levels – encoding a protein that inactivates pyruvate dehydrogenase and hence the initial step in mitochondrial (glycolysis to citrate cycle) oxidation – was higher in F adipose tissue (Figure 4). Indeed, *Pdk4* was higher across all 3WATs in F adipose tissue (3WAT averaged mean intensity fold-change: 2.6) but not in liver muscle or kidney, exemplifying a secondary (non-QTL) but adipose-specific change in adipose tissue intermediary metabolism. *Ldh2* encoding lactate dehydrogenase (pyruvate to lactate for *de novo* lipogenesis) showed a similar a 3WAT-specific increase (mean intensity fold-change of ∼5) in F adipose tissue, consistent with a major, though likely secondary role for genes of intermediary metabolism in the development of obesity.

### Line and Diet Effects on angiogenic/hypoxia genes

Key changes were observed in the angiogenesis pathway (*Angpt1*, *Angptl4* and *Anglr1*, *Hif1a*, *Apln*, *Il8*) in F adipose tissue (Figure 5 and microarray) consistent with adipose tissue hypoxia being an early molecular link between obesity and insulin resistance [32]–[34]. Distinct matrix metalloproteases associated with the remodelling of adipose tissue [35] were selectively elevated in F (*Mmp3*, *Mmp12*) or L (*Mmp9*) adipose tissue (Figure 5 and microarray). Thus, alternate inflammatory, angiogenic and adipose remodelling mechanisms are active in the two lines that may impact upon the severity of metabolic disease.

### Functional validation of key divergently expressed genes

**Figure 6:**
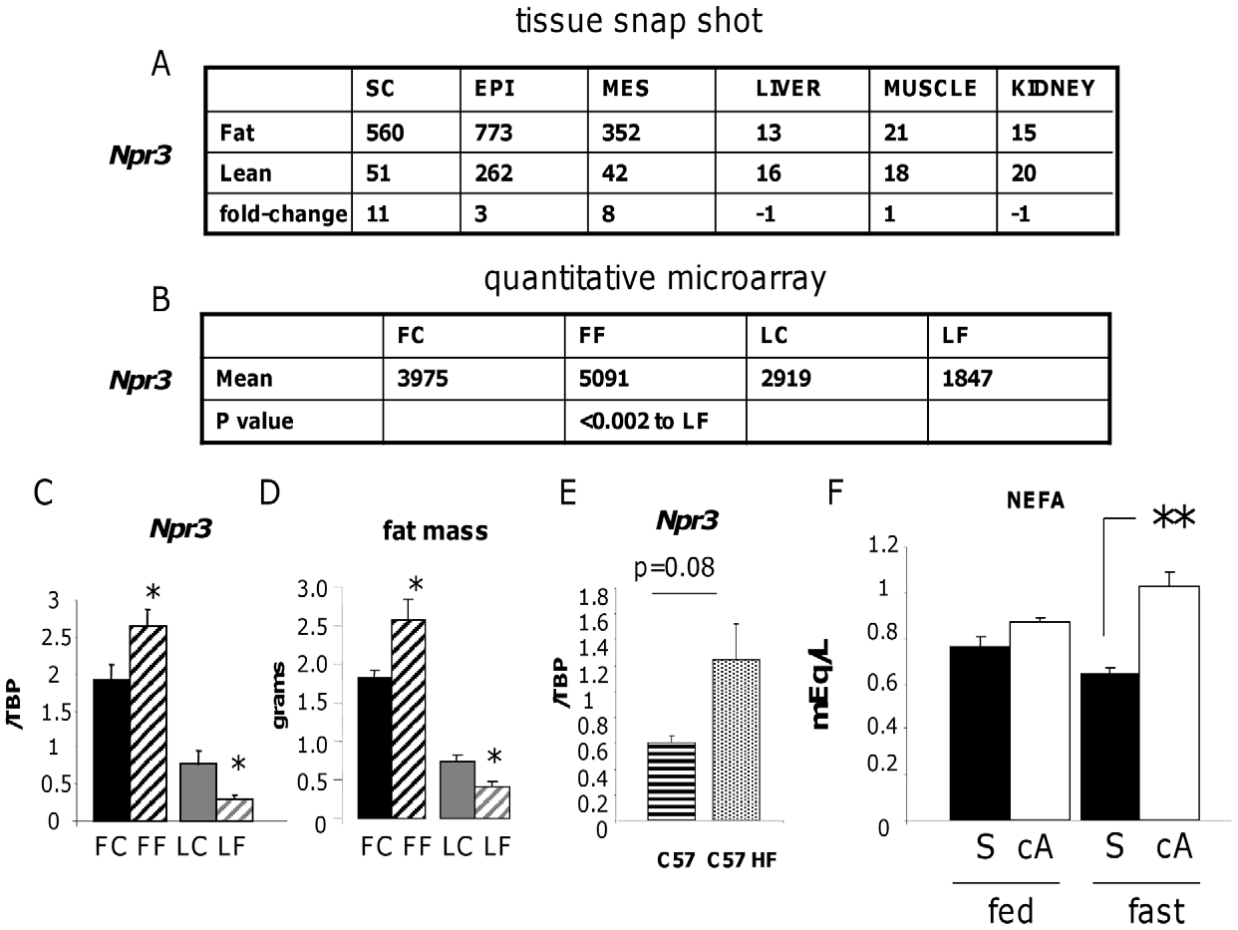
Validation and functional investigation of the candidate obesity gene *Npr3*. **A.** Snap-shot pooled transcriptome microarray chip intensities (arbitrary units) across subcutaneous (SC), epididymal (EPI), mesenteric (MES) adipose tissues versus liver, muscle and kidney in Fat (F) and Lean (L) mice with the rounded expression ratio in bold. **B.** Mean chip intensity from quantitative microarray (n = 4) in Fat and Lean mice fed control (FC, LC) or high fat diets (FF, LF) with relevant P value for significant differences. **C.** RT-PCR validation of changes in *Npr3* gene expression along with its relationship to **D.** Altered fat mass in response to high fat diet in subcutaneous adipose tissue of control diet-fed Fat (FC, black bars), high fat diet-fed Fat (FF, black hatched bars), control diet-fed Lean (LC, grey bars) and high fat diet-fed Lean (LF, grey hatched bars) mice. * = P<0.05 significant effect of diet within a line. **E.** Expression levels of *Npr3* in subcutaneous fat of control diet (horizontal hatched bar) or HF fed (heavily stippled bar) C57BL/6J mice (C57). **F.** The effects of fasting (fast) on plasma free fatty acid levels (NEFA) in C57BL/6J mice implanted with minipumps releasing saline (S) or 100 ng/day cyclic ANP3-23 (cA). ** = P<0.01 for an effect of cA treatment of fasting NEFA.

**Figure 7:**
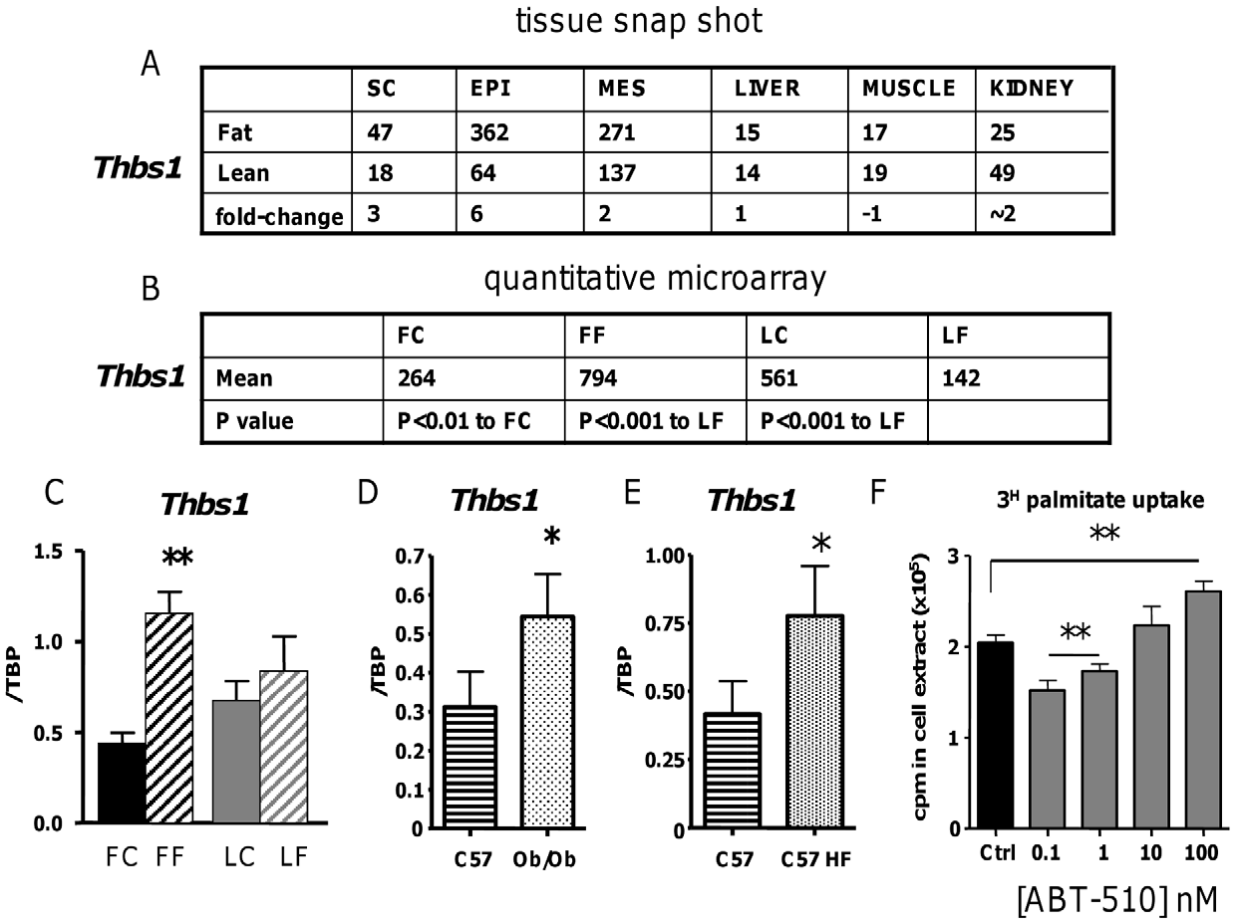
Validation and functional investigation of the candidate obesity gene *Thbps1*. **A.** Snap-shot pooled transcriptome microarray chip intensities (arbitrary units) across subcutaneous (SC), epididymal (EPI), mesenteric (MES) adipose tissues versus liver, muscle and kidney in Fat (F) and Lean (L) mice with the rounded expression ratio in bold. **B.** Mean chip intensity from quantitative microarray (n = 4) in Fat and Lean mice fed control (FC, LC) or high fat diets (FF, LF) with relevant P value for significant differences. **C.** RT-PCR validation of changes in *Thbps1* gene expression in subcutaneous adipose tissue of control diet-fed Fat (FC, black bars), high fat diet-fed Fat (FF, black hatched bars), control diet-fed Lean (LC, grey bars) and high fat diet-fed Lean (LF, grey hatched bars) mice. ** = P<0.01 versus FC. **D.** *Thbps1* mRNA levels in control-fed C57BL/6J (C57, horizontal striped bars) and genetically-obese leptin-deficient Lep^ob^ mice (Ob, lightly stippled bars) and **E.** *Thbps1* mRNA levels in control-fed C57BL/6J (C57, horizontal striped bars) and high fat diet fed C57BL/6J mice (C57 HF, heavily stippled bars). * = P<0.05 versus lean control. **E.** The effects of the thrombospondin-1 type 1 repeat ABT-510 (0.1–100 nM, grey bars) on ^3H^-palmitate uptake into fully differentiated 3T3-L1 adipocytes. ** = P<0.01 versus control (Ctrl, black bar), non-ABT510 treated cells.

**Figure 8:**
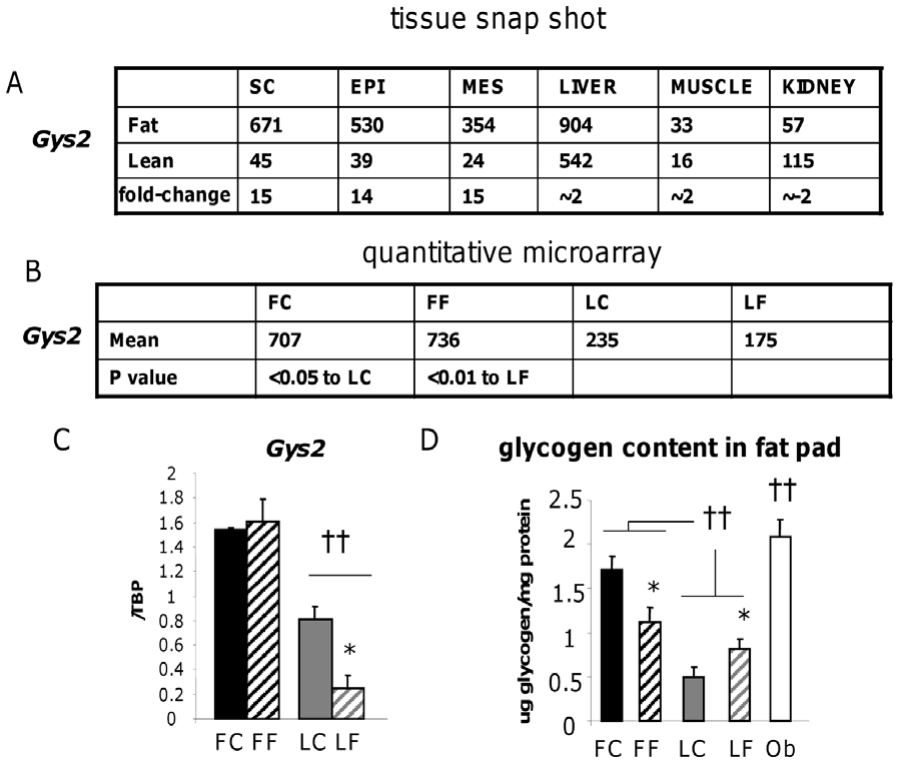
Validation and functional investigation of the non-QTL linked, obesity-associated gene *Gys2*. **A.** Snap-shot pooled transcriptome microarray chip intensities (arbitrary units) across subcutaneous (SC), epididymal (EPI), mesenteric (MES) adipose tissues versus liver, muscle and kidney in Fat (F) and Lean (L) mice with the rounded expression ratio in bold. **B.** Mean chip intensity from quantitative microarray (n = 4) in Fat and Lean mice fed control (FC, LC) or high fat diets (FF, LF) with relevant P value for significant differences. C. RT-PCR validation of changes in *Gys2* gene expression in subcutaneous adipose tissue of control diet-fed Fat (FC, black bars), high fat diet-fed Fat (FF, black hatched bars), control diet-fed Lean (LC, grey bars) and high fat diet-fed Lean (LF, grey hatched bars) mice. †† = P<0.01 difference between F and L lines, * = P<0.05 indicates and effect of diet within the L line. **D.** Glycogen content of fat pads in subcutaneous adipose tissue of control diet-fed Fat (FC), high fat diet-fed Fat (FF), control diet-fed Lean (LC) and high fat diet-fed Lean (LF) mice and in genetically-obese leptin-deficient Lep^ob^ (Ob, white bars) mice. †† = P<0.01 difference between F or Ob versus and L mice and * = P<0.05 indicates and effect of diet within Fat or Lean lines.

To further validate our stratified obesity gene enrichment approach we investigated the functional impact of 3 genes implicated in F-line obesity on fat cell function. We chose natriuretic peptide receptor C (*Npr3*), a gene associated with the *Fob3b* QTL (Figure 6) and thrombospondin-1 (*Thbs1*; Figure 7) a *Fob1* QTL-associated gene as candidates that had passed all the selection criteria. We also investigated one non-QTL associated gene, glycogen synthase 2 (*Gys2*; Figure 8) as it had passed all but one (within QTL) of our stringent adipose enrichment criteria. Although more likely a secondary gene response, the conspicuously large fold-change (15, versus 6 and 4 for *Npr3* and *Thbs1*, respectively), supporting evidence from functionally related gene expression changes (*Pdk4*, *Ldh2*) and the additional possibility that *Gys2* may fall within an un-characterised minor QTL, we wished to maintain scientific balance in our approach by including it.

### Investigation of *Npr3* uncovers of a novel role for the ANP system in rodent lipolysis *in vivo*

Natriuretic peptide receptors (NPRs) regulate blood pressure and are particularly relevant to exercise-induced lipid mobilisation [36]. The receptor encoded by *Npr3* is a signalling deficient isoform, highly expressed in adipose tissue and kidney which is involved in clearance of active atrial natriuretic peptide (ANP). *Npr3* was elevated in all 3 fat depots of F mice in the snap-shot array and in the quantitative array (Figure 6A–B). RT-PCR validation showed that *Npr3* expression (Fig. 6C) closely followed the changes in subcutaneous adipose tissue mass in response to HF feeding in F and L mice (Figure 6D) and showed a trend towards being increased in 4 week HF-fed C57BL/6J mice (Figure 6E). ANP was shown to induce lipolysis in primate, but not rodent, adipocytes through activation of the NPRA (*Npr1*) [37]. We investigated the effects of the NPR3-selective agonist cyclic ANP (cANP) on lipolysis *in vivo* by implanting cANP-releasing minipumps into C57BL/6J mice, a standard model of high fat-diet induced obesity. Plasma ANP levels were elevated in cANP-treated mice (saline, fed state: 17±3 ng/ml versus cANP infusion, fed state: 125±21 ng/ml, P<0.001), as expected from its blockade of *Npr3*-mediated ANP clearance [38]. This did not affect body weight gain with short-term (4 week) HF feeding (data not shown). To test if the ANP system might be involved adipocyte lipolysis we challenged the mice with a 24 hour fast during week 3 of the HF experiment. Fasting glucose levels were in the normal range for loss of an overnight feeding bout (saline treated: 6.1±0.4 mmol/L, cANP treated: 5.8±0.2 mmol/L). We found a novel effect of fasting to elevate endogenous ANP levels. This was exaggerated in cANP-infused mice (saline fasted state: 61±11 ng/ml, cANP fasted state: 436±54 ng/ml, P<0.001). In addition, cANP-treated mice showed a significant increase in fasting NEFA levels that was not apparent in the fed state (Figure 6F). Our data suggest that fasting is permissive for a physiological lipolytic effect of ANP in rodents which is exaggerated by blocking ANP clearance with cANP *in vivo*. High NPR3 levels in adipose tissue of Fat mice may abrogate the lipolytic effect of ANP locally through increased clearance of lipolytic ANP and thus contribute to obesity. The lipolytic effect of ANP may only be manifest in states of low insulin action such as fasting or, perhaps more relevant, in combination with other factors (eg obesity) that contribute to pre-existing insulin resistance [13].

### Functional insight into a novel obesigenic role for elevated *Thbs1* on adipocytes

Control of vascular growth during adipose tissue expansion is a major regulator of adiposity [39]. We noted that a number of genes in the angiogenic/angiostatic cascade were differentially expressed between the F and L lines (Figure 5 and microarray). Thrombospondin1 (*Thbs1*) was elevated across the three fat depots in the snap-shot analysis and this was confirmed in the quantitative microarray (Figure 7 A–B). *Thbs1* is positioned in the *Fob1* QTL [5]. During the course of validation of this array work, others published [40] that thromobospondin-1 was elevated in obesity and was an adipocyte-derived cytokine (adipokine). Although realtime PCR analysis did not show any *Thbs1* change in L adipose tissue (not validated) the increase of *Thbs1* in F mice was validated (Fig. 7C). *Thbs1* mRNA was also elevated in adipose tissue from HF-induced- and genetically-obese mice (Figure 7D–E). Critically, the angiostatic peptide fragment [41] of thrombospondin-1 (ABT-510) had distinct concentration-dependent effects, reducing fatty acid uptake into 3T3-L1 adipocytes *in vitro* at low concentrations (0.1–1 nM) but increasing fatty acid uptake at high physiological (100 nM) concentrations (Figure 7F). Our data suggest Thrombospondin-1 may have a dual effect. In the adipose vasculature it may curtail angiogenesis [41] and potentially prevent fatty acid uptake through its CD36 cell surface receptor [42] at low concentrations. However, at high concentrations thrombospondin-1 may have a direct hypertrophic effect on adipocyte lipid accumulation thus promoting obesity.

### Elevated *Gys2* is associated with abnormal carbohydrate metabolism and increased glycogen deposition in adipose tissue

Despite not being positioned within any of the 4 major QTLs, *Gys2* gene mRNA was markedly higher (∼14-fold) selectively in F adipose tissues in the snap-shot experiment and this was confirmed with the quantitative microarray and RT-PCR validation (Figure 8A–C). Glycogen content was also elevated in the F adipose tissue and in *Lep^ob^* mice, a monogenic model of morbid obesity (Fig. 8D) suggesting ectopic glycogen is a feature or adipose tissue in obesity. Notably, mRNA levels of the regulatory subunit of phosphatase 1 (*Ppp1r3d*) that targets glycogen synthase is one of the novel *Fob1* obesity candidate genes (Table 3). This finding is consistent with a switch in intermediary metabolism from carbohydrate oxidation (eg *Pdk4*) to inefficient lipid oxidation which leads to a net increase in lipid synthesis (eg *Ldh2*; lactate, driving glyceroneogenesis) and ectopic carbohydrate storage in adipose tissue. Of note, glycogen complexes with water and thus has a higher mass per mole than lipid. Ectopic glycogen deposition may therefore make an important contribution to fat pad mass per unit stored.

### Conclusions

We have gained insight into metabolic, inflammatory and angiogenic remodelling responses characteristic of obesity, some of which overlap with those described by others [43]–[45] and that are consistent with broad changes found in microarray studies on adipose tissue from obese rodents and humans [46]–[52]. Crucially, some of our observations are unique to our model. This is very likely due to the polygenic nature and unique selection-based origin of the divergent lines which reveals obesity (and indeed leanness)-related changes beyond other commonly used models (eg monogenic Lep^ob^ or HF-fed C57BL/6J), with relevance to human obesity and its associated metabolic disturbances. Devising a stratified systems approach integrating gene expression, tissue-specific, depot-specific and functional data with positional (QTL) information allowed a more rigorous test to identify and validate candidate obesity genes. We have been able to show that a prime obesity candidate gene (*Npr3*), that fulfilled all the required criteria, turned out to have a complex, context-dependent effect on fat mobilisation in mice *in vivo*. *Npr3* may also link obesity-related hypertension [13] in the model. Our approach has led to identification of a novel direct effect of thrombospondin-1 on fat cell hypertrophy. The exact mechanism whereby *Thbs1* promotes obesity will require further study due to its complex modular structure, functions and multiple receptors [53]. Nevertheless the effect of the type 1 repeat of thrombospondin-1 (ABT-510) suggests a functional cross-talk between adipose tissue endothelial cells and fat cells. Given the success of our stratified enrichment strategy in linking candidate genes with fat cell function we anticipate that the other QTL-associated genes will also have direct and functionally relevant effects on obesity. This is further emphasised (see Table 3) by the reported (*Np3r*, *C1qr*) or inferred roles of these genes in either fat cell function (*Ttc7b*, *Tuba1*) or obesity-associated insulin resistance (*Ppp1r3d*, *Trp53inp2*, *Fgf13*, *Fmr*).

Our results highlight the benefit of using transcriptomics in addition to F_2_ QTL information to identify secondary, adipose-enriched genes (*Gys2*, *Pdk4*, *Ldh2*; intermediary fat and carbohydrate metabolism in fat cells) in parallel with positional candidate genes. These genes likely contribute to obesity (eg ectopic glycogen storage) and its consequences regardless of the mechanistic origin of excessive weight gain. The original QTL mapping study [5] was done using the outbred F and L lines and a low-density genetic marker spacing with the power to detect only major QTL effects. Our current study may therefore have uncovered novel minor QTLs that were here regarded as ‘non-QTL-associated’, and so it was important to document a prime example of at least one gene (*Gys2*) that otherwise, and rather conspicuously (large fold change across 3WATs, validated in quantitative array), passed our filtering criteria. Potentially, *Gys2* or other genes could be confirmed as true expression (e)QTLs in future high resolution F_2_ mapping. Of further note, many of the inflammatory changes are non-QTL associated, suggesting that these genes are secondary, though still important, responders to obesity. Interestingly, there were no major expression differences in well-characterised transcriptional pathways regulating adipocyte formation (C/EBPs, PPARs, GATAs, RIP140, ERRs, KLFs, WNTs, STATS, E2Fs, Dlk, Dlp, Foxos, PRDM16), or fat accumulation and synthesis (SREBPs).

The L mice remarkably lose fat mass with HF feeding and may model beneficial adipose tissue changes with weight loss [13]. After bariatric surgery in obese patients there is a switch in gene expression from prominent stress-related pathways to genes consistent with remodelling of the adipose tissue [54]. L mice show a similar response with alternate-inflammatory, adipose remodelling and lipolytic effects alongside improved metabolic function with HF diet [13]. Thus the gene pathways changing in L mice losing fat mass with HF feeding are not akin to a ‘lipodystrophic’ re-distribution of calories into non-adipose tissues with consequent worsening of metabolic disease. This data may point to important leanness genes that act independently in adipose tissue to help cope with cellular stress and this is an area of important future investigation.

Future work in the adipose tissues of refined congenic lines with discrete QTLs introgressed on the comparator genetic background will help apply additional filters for gene expression and functional characterisation of candidate adiposity genes. This approach has been successful in identifying *Pc2* as an important obesity candidate gene, albeit of hypothalamic origin, in a C57BL/6J subcongenic line carrying a 7.4 Mb region of chromosome 2 from SPRET/Ei mice [55]. A similar approach led to the positional cloning and identification of the *Prcp* gene as being causal for a hypothalamic mechanism of leanness involving α-MSH degradation [56]. An optimal test to prove that a candidate gene is causal for the QTL effect is quantitative complementation [57], which requires knockout models in particular genetic backgrounds. These resources are currently unavailable for the F and L lines. Therefore, the approach of developing novel subcongenic lines with ever smaller donor segments combined with bioinformatics, sequence, expression, and functional analyses remains an optimal strategy to find causal sequence variation for obesity candidate genes identified in this study. Recent SNP genome-wide association studies (GWAS) in humans revealed that quantitative variation in obesity is due to the action of numerous QTLs of relatively small effect; each of the obesity loci detected generally account for less than 1% of the phenotypic variance [1]–[2], [12]. In an extremely large scale GWAS study exploring a well characterised quantitative trait, height, the 180 loci uncovered accounted for only 10% of phenotypic variation [58]. Quantitative traits like obesity are therefore controlled by many more genes than initially predicted and highly statistically-powered animal studies in defined models such us the one used here are likely to uncover additional obesity loci that would remain undetected in human GWAS.

The unique selection basis of the Fat and Lean strains on fat pad mass divergence highlights some previously unidentified molecular mechanisms contributing to fat mass accumulation and its downstream metabolic sequelae that may be amenable to therapeutic intervention. The genes and pathways identified by our stratified enrichment approach may prove important over and above the genetically determined appetitive, energy expenditure and activity-mediated drivers of fat mass in rodents and humans.

## Methods

### Experimental Design

#### Experiment 1. ‘Snap-shot’ pooled transcriptome microarray

Our first experiment was designed to look across a panel of tissues of the F and L mice including 3 white adipose tissue depots, liver, muscle and kidney for broad and large qualitative fold-changes in gene expression (Figure 1). Individual tissues were pooled from 3 chow fed mice of each line (ie 3xSC, 3xEPI, 3xMES, 3xliver, 3xmuscle and 3xkidney samples were combined to produce representative distinct tissue RNA with reduced biological variability in this initial step). This pooled transcriptome approach is referred to as the ‘snap-shot’ approach. Before microarray we confirmed that previously described changes in leptin and 11β-HSD1 expression [13] were found in the individual adipose tissues depots of the sampled mice by northern blot (data not shown). RNA was hybridised to Affymetrix Genechip 2.0 arrays according to standardised protocols at the (Ark Genomics, Roslin, UK). We again used previously described differences in gene expression [13] as validatory transcriptome ‘landmarks’ for the qualitative microarray data. The snap-shot approach allowed us to 1. Assess which genes were grossly different between the Fat and Lean lines across all tissues tested. 2. Provide information on which genes were divergently expressed selectively across all white adipose depots. 3. Apply a stricter criterion for genes that were specifically altered in the 3 white fat depots but not in the other metabolic tissues to increase the likelihood of identifying adipose-specific causal obesity genes. Note the original selection criterion of the F and L mice was on divergent fat pad mass and that the obesity is not the result of increased food intake [6], [7]. Moreover, this was useful since the mixed genetic background of the base population may have carried ‘bystander’ genes that are differentially expressed between the lines in both adipose and non-adipose tissues, but that are not related to the divergent obesity and metabolic phenotype. 4. Obtain information on possible adipose tissue *depot-specific* changes in gene expression that are informative as regards the impact of different fat depots on metabolic disease.

#### Experiment 2. Exon-chip microarray with dietary intervention:

Our second experiment was designed to look at the adipose tissue depot which showed the greatest divergence in mass between the lines in response to chronic high fat feeding [13]. We took subcutaneous fat from control diet and high fat fed F and L mice after 18 weeks on the diets (n = 4) which allowed us to 1.Validate changes in subcutaneous fat from experiment 1 and 2. Perform quantitative analyses on the changes in gene expression between the lines and identify line-specific effects of the diet.

#### Bioinformatics analysis of microarray data

1. Experiment 1 looked at qualitative fold changes in gene expression between the lines in the 3 WAT depots (subcutaneous (SC), epididymal (EPI) and mesenteric (MES), liver (L), muscle (M) and kidney (K). We set the fold-difference threshold for changes of interest to >±1.5 (where denoted a ‘−’ refers to genes that are up regulated in the Lean line and, positive numbers denotes genes that are up regulated in the F lines, respectively). Initially we did not specify any range limitations for gene expression changes in L, M or K. A second filter set the fold change within L, M or K to be within <±1.5. This search narrowed our targets to white adipose tissue-specific changes in gene expression, with the caveat that many gene pathways maybe linked between or operate differently/reciprocally from white fat and non-adipose tissues. An example would be the opposing effects of glucocorticoids in adipose versus liver for 11β-HSD1 expression [13]. Further, for candidate genes we excluded all genes whose absolute signal was below the threshold of 100 (genes unlikely to be meaningfully expressed in the adipose) in one or both lines, with the caveat that it is possible some genes may be effectively switched on or off in adipose tissue of one or other line (eg *Depdc6* and *Gsn*, see text).

2. Quantitative microarray analysis of subcutaneous fat in experiment 2 looked at fold changes in gene expression in a single fat depot, but allowed us to further assess the effects of chronic high fat feeding between and within the lines. We set the fold difference threshold to ±1.5 as before.

#### QTL-informed analysis

Analysis of both experiment 1 and experiment 2 benefited from the previously identified major QTL information [5], which we introduced as a filter to provide more information on which genes were more likely to represent causal genes (found within the 95% confidence interval of the four major QTL boundaries) and those which were more likely secondary (not found within the 95% confidence interval of the four major QTLs).

#### Validation

Our validation steps consisted of realtime PCR (RT-PCR) for ‘landmark’ genes on RNA from adipose tissues, functional assessment of biochemical changes (glycogen) in the adipose tissue of the lines and a number of *in vitro* and *in vivo* studies to test distinct hypotheses (Natriuretic peptide receptor and thrombospondin1).

### F and L mice

#### Ethics Statement

All animal experiments were performed according to local ethical guidelines of The University of Edinburgh Ethics Committee and those of the (Scientific Procedures) Act (1986) of the UK Government Home Office under the auspices of an approved Home Office Project License (60/3962).

The long-term selection and further development of the F and L lines and details of the genetic basis of the line divergence and the inbreeding period are described elsewhere [4]–[7], [15]–[16]. Mice derived from the inbred lines were used in this study [4]. Extensive characterisation of our lines in previous studies determined that body weight gain is highly correlated with fat mass accretion in fat mice [4]–[7]. This was substantiated in several genetic mapping experiments [5], [15]–[16] demonstrating that the fat% trait and body weight trait co-localise to the same QTL regions with significant LOD scores. We followed bodyweight change closely and interpreted the changes in our lines as being primarily due to altered body fat mass. Animals were fed on pelleted Rat and Mouse breeder and grower diet (Special Diets Services, SDS, UK Ltd., Witham, Essex, UK) or with defined low (11% calories as fat with sucrose; D12329) and high fat (58% calories as fat with sucrose; D12331) diets (Research Diets, New Brunswick, New Jersey).

### Tissue and plasma measurements

At the end of the experiment, mice were killed within 1 minute of disturbing the home cage by cervical dislocation, to avoid stress-induced changes in metabolic parameters. Blood was collected in EDTA coated tubes (Sarstedt, Numbrecht, Germany). Plasma insulin was measured by ELISA (Crystalchem, Downers Grove, IL, USA), glucose by (Infinity reagent, Sigma, Dorset, Uk) and free fatty acids (Wako Diagnostics, Neuss, Germany) levels (FFA) were measured with a colorimetric method. Liver (L), muscle (quadriceps, M), kidney (K), and epididymal (E), subcutaneous (S), and mesenteric (M) adipose tissues were collected, weighed and stored at −80°C.

### RNA extraction and analysis

RNA was extracted using 800 µl TRIzol reagent (Invitrogen, Paisley, UK) per 50 mg tissue. Briefly, 200 µl of chloroform was added to the homogenate then centrifuged at 12,000 rpm for 1 min to remove cell debris. The supernatant was then vortexed for 15 s and centrifuged at 12,000 rpm for 15 min at 4°C. The upper (aqueous) layer was removed, mixed with 30 µl of RNAid+ matrix (Anachem, Luton, UK) and agitated for 5 min before centrifugation at 12,000 rpm for 1 min. The supernatant was removed and washed 3 times with 500 µl of RNA wash (Anachem, Luton, UK), resuspended in 20 µl of DEPC-treated water with 10 mM DTT, 1 U/ml RNasin (Promega, Southampton, UK) and eluted by incubation at 55°C for 10 min. Concentration and purity of RNA was assessed using a GeneQuant RNA/DNA calculator (GE healthcare, Amersham, UK) before northern blot or real-time PCR analysis. For Northern blotting, 5 µg of RNA was denatured at 65°C for 15 min in a mixture of MOPS, deionised formamide and formaldehyde as described (Morton et al., 2005) and run on denaturing MOPS/formaldehyde 0.8% agarose gels. Briefly, RNA was transferred using capillary action onto a nylon membrane for hybridisation, using 20× SSC as the transfer buffer. RNA was crosslinked by UV exposure (Spectronics Corporation, power at 1200×100 µw/cm^2^). Membranes were prehybridised at 65°C for 3 h with 18 ml of phosphate buffer and 9 ml of 20% SDS in a hybridization bottle. 1 ml of salmon testes DNA (10 mg/ml) was denatured at 100°C for 10 min and added to the pre-hybridization mix. Radiolabelled cDNA probes were made for genes of interest using a rediprime2 random prime labelling kit (GE Healthcare). 25 ng DNA template (PCR fragment) was diluted to 45 µl with TE buffer. The probe was then denatured at 100°C for 15 min and immediately cooled on ice for a further 10 min. Denatured DNA was then added to the reaction tube together with 5 µl of [^32^P]-dCTP. The reaction was incubated at room temperature for 2 h then labelled cDNA was purified through a Nick column (GE healthcare, Buckinghamshire UK). The hybridization mixture was then incubated overnight at 55°C. Three 15 minute 50 ml washes were performed; an initial wash at room temperature with 2×SSC and 0.1% SDS was followed by 2 washes at 65°C, with 1×SSC, 0.1% SDS then 0.5×SSC, 0.1% SDS. The washed membrane was wrapped in Saranwrap and exposed to a phosphorimager screen (Fujifilm, Bedford, UK) for 10 min and scanned using a Fuji BAS phosphorimager. Transcipt levels were quantified using Aida software (Advance image data analyzer Version 3.44.035).

### Microarray analysis

For the snapshot experiment, tissue RNAs were prepared using Qiagen RNeasy kits, processed through standard Affymetrix protocols, and hybridized to Affymetrix Mouse Genome 430 2.0 GeneChips (n = 4 per group, Affymetrix, Santa Clara). Raw CEL file data were imported into BioConductor for background subtraction and normalization with the Robust Multichip Average (RMA) algorithm. Differential expression was determined using the Bioconductor Limma tool and the Benjamini and Hochberg FDR method. Annotation data for the genes were obtained from NetAffx. WebGestalt (http://bioinfo.vanderbilt.edu/webgestalt, Vanderbilt University, USA), DAVID (http://david.abcc.ncifcrf.gov/, National Institute of Allergy and Infectious Diseases) were used to cross validate clustering and pathways analyses. Full microarray data are MIAME compliant and are available in the ArrayExpress database under the accession number M-EXP-3091. For the quantitative microarray RNA was prepared with Qiagen RNeasy kits and hybridised to Affymetrix mouse ST 1.0 GeneChips. Data were imported into the Onechannelgui package of Bioconductor, normalised with RMA (with background subtraction) and analysed with Limma with Benjamini and Hochberg FDR multiple testing correction. Data are available in ArrayExpress with accession number E-MEXP-3094. Microarray processing was by the ARK Genomics team at the Roslin Institute.

### Real-time PCR

cDNA was synthesised from 2 µg RNA using Reverse Transcription system (Promega, Southampton, UK) with oligo(dT) primer, according to the manufacturer's instructions. Gene-specific mRNA levels were measured using the LightCycler® 480 Real-Time PCR system (Roche Diagnostic Ltd, West Sussex, UK) with light cycler 480 probe master (Roche Diagnostic Ltd, West Sussex, UK) and TaqMan® Gene expression Assays (Applera, Cheshire, UK). Samples were analysed in triplicate with each PCR reaction containing 4.5 µl cDNA, 5 µl master mix and 0.5 µl primer-probe. Results are expressed as the ratio of gene of interest corrected to the housekeeping gene TBP mRNA levels as an internal control. TaqMan® Gene expression Assays were inventoried and are available from the Applera website (http://www.appliedbiosystems.com/absite/us/en/home/applications-technologies/real-time-pcr.html).

### 3T3-L1 cell culture

The 3T3-L1 cell line is a preadipocyte cell line derived from the Swiss 3T3 mouse fibroblast cell line [59] Cells were cultured in Dulbecco's modified Eagle medium (DMEM) (Cambrex, Verviers, Belgium) supplemented with 10% new born calf serum (NCS), 2 mM L-glutamine, penicillin (50 U/ml) and streptomycin (50 ug/ml) (Invitrogen, Paisley, UK) at 37°C in humidified atmosphere with 5% CO2. Confluent 3T3-L1 cells were subjected to differentiation protocol. Maintenance medium was changed at day 0 to differentiation DMEM but with additional 10% FBS to replace NCS and supplemented with 0.5 mM isobutylmethylxanthine (IBMX), 0.25 µM dexamethasone (Dex), 1 ug/ml insulin and 100 nM Rosiglitazone.

### Palmitate uptake

Pre-adipocytes (passage 8–11) were seeded onto 12 well plates at a density of 2.5×10^5^ cells/well. Adipocytes were then fully differentiated with the above protocol and incubated with charcoal-stripped fetal calf serum overnight. ABT-510 1–100 nM (dissolved in sterile PBS) was incubated in stripped serum with the adipocytes for 6 hours at 37°C. Palmitate uptake was measured by the addition of unlabeled palmitate, dissolved in ethanol, and tracer (final 200 nM) radiolabelled ^[3H]^ palmitate (0.3 µCi) in a 0.1% BSA solution. Cell-associated radioactivity was obtained by counting aliquots of both the medium and in the cells scraped with 10% SDS in 2 mls of aqueous scintillation fluid (GE healthcare) in a Beckman LS330 scintillation counter.

### Cyclic atrial natriuretic peptide (cANP) studies *in vivo*

13 C57/BL6J male (in house colony) at approximately 4-month of age were used for the study. Mice were weighed and divided into two groups to match initial body weight. Allowing for variations in body weight, mini pumps were prepared using sterile saline or varying amounts of cANP3-23 (Bachem, Switzerland) dissolved in sterile saline to allow infusion of ∼100 ng/day cANP3-23 a dose chosen to cause maximal NP3R occupancy [38]. The pumps were primed in saline and stored in the refrigerator for three days prior to implantation. On the day of surgery, animals were fasted for 4 hours, weighed and blood samples were taken from tail nicks to measure basal ANP, glucose and circulating FFAs. Animals were anaesthetised with 5% isofluorane/oxygen mixture and the pumps were inserted subcutaneously. After recovery, animals were started on a 58 kcal% fat w/sucrose high fat diet (Research Diets). Body weight and food consumption were measured routinely twice a week. At week 3, a physiological challenge to elevate free fatty acids was performed (24 hour fast) and the change in body weight recorded to determine fat mobilisation.

### Glycogen assays

Tissue glycogen was assessed using a modified acid hydrolysis extraction protocol [60]. Briefly, 500 µL 0.03 m HCl were added to approximately 200 mg of adipose tissue, homogenized and then boiled for 45 min at 80–90°C in a water bath. Sample pH was adjusted to pH 5 by adding a small amount of either 1.1 M HCl. The samples were incubated water bath shaker at 37°C for at least 2 h. Glucose levels were determined at room temperature as described above.

### Statistical analyses

Gene expression differences in validation realtime was analysed using 2-way ANOVA for line and diet effects followed by post-hoc Holm-Sidak multiple comparison tests using Sigmastat version 3.5 (Systat Software Inc). Effects of treatments on biological parameters such as fatty acids, tissue glycogen or palmitate uptake were assessed using 1-way ANOVA. P-values below 0.05 were accepted as statistically significant.

## Supporting Information

Table S1 Selected strains with high fasting plasma glucose levels after chronic HF feeding.
(DOC)

Figure S1 Toll-like receptor 13 (*Tlr13*) mRNA levels in adipose tissues from obese diabetic mouse strains. The bars show the relative expression of *Tlr13* in the adipose tissues from multiple strains (http://biogps.gnf.org). *Tlr3* expression patterns are shown from 2 eQTL analyses (gnf1m32524_at and 1457753_at) from the gene expression/acitivty chart of biogps from ‘Fat’ or ‘Adipose’. High *Tlr13* expression is found in mouse strains that are also obese and diabetic (Supplemental Table S1 [70]).
(TIFF)
