# Supplementary material for: A Stratified Transcriptomics Analysis of Polygenic Fat and Lean Mouse Adipose Tissues Identifies Novel Candidate Obesity Genes
Source: PLoS One. 2011 Sep 7;6(9):e23944. doi: 10.1371/journal.pone.0023944 (PMC3168488; doi:10.1371/journal.pone.0023944)
Supplement: Table S1 — Selected strains with high fasting plasma glucose levels after chronic HF feeding. (DOC) [file pone.0023944.s001.doc]

**Supplemental Table 1.** **Selected strains with high fasting plasma glucose levels after chronic HF feeding.** Measurements are [plasma glucose (mg/dL) after a 4h fast with 17 weeks of high-fat diet 17wks](javascript:;)  [70].

| Strain | Sex | Strain/sex mean value | SD and N | Z-score |
| --- | --- | --- | --- | --- |
| [NOD/ShiLtJ](javascript:;) | male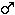 | 348 | ±237   N=6 | 4.12   43 strains |
| [SPRET/EiJ](javascript:;) | female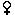 | 266 | ±22.5   N=4 | 3.66   43 strains |
| [NON/ShiLtJ](javascript:;) | male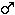 | 232 | ±182   N=12 | 1.53   43 strains |
| [SPRET/EiJ](javascript:;) | male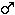 | 231 | ±37.7   N=5 | 1.50   43 strains |
| [C57BL/10J](javascript:;) | female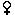 | 196 | ±34.1   N=9 | 1.47   43 strains |
| [MSM/Ms](javascript:;) | female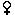 | 191 | ±32.1   N=8 | 1.31   43 strains |
| [C57BL/6J](javascript:;) | female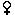 | 190 | ±24.0   N=10 | 1.28   43 strains |
| [MSM/Ms](javascript:;) | male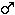 | 216 | ±41.2   N=11 | 1.17   43 strains |
| [CBA/J](javascript:;) | female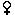 | 185 | ±36.5   N=8 | 1.12   43 strains |
| [KK/HlJ](javascript:;) | female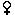 | 179 | ±18.2   N=7 | 0.93   43 strains |
| [FVB/NJ](javascript:;) | female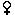 | 175 | ±25.6   N=8 | 0.81   43 strains |
| [C57BL/6J](javascript:;) | male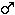 | 199 | ±31.5   N=10 | 0.79   43 strains |
| [SM/J](javascript:;) | male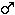 | 198 | ±41.5   N=9 | 0.77   43 strains |
| [C57BR/cdJ](javascript:;) | female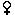 | 173 | ±23.6   N=10 | 0.75   43 strains |
