# Supplementary figures and images for: A Stratified Transcriptomics Analysis of Polygenic Fat and Lean Mouse Adipose Tissues Identifies Novel Candidate Obesity Genes
Source: PLoS One. 2011 Sep 7;6(9):e23944. doi: 10.1371/journal.pone.0023944 (PMC3168488; doi:10.1371/journal.pone.0023944)

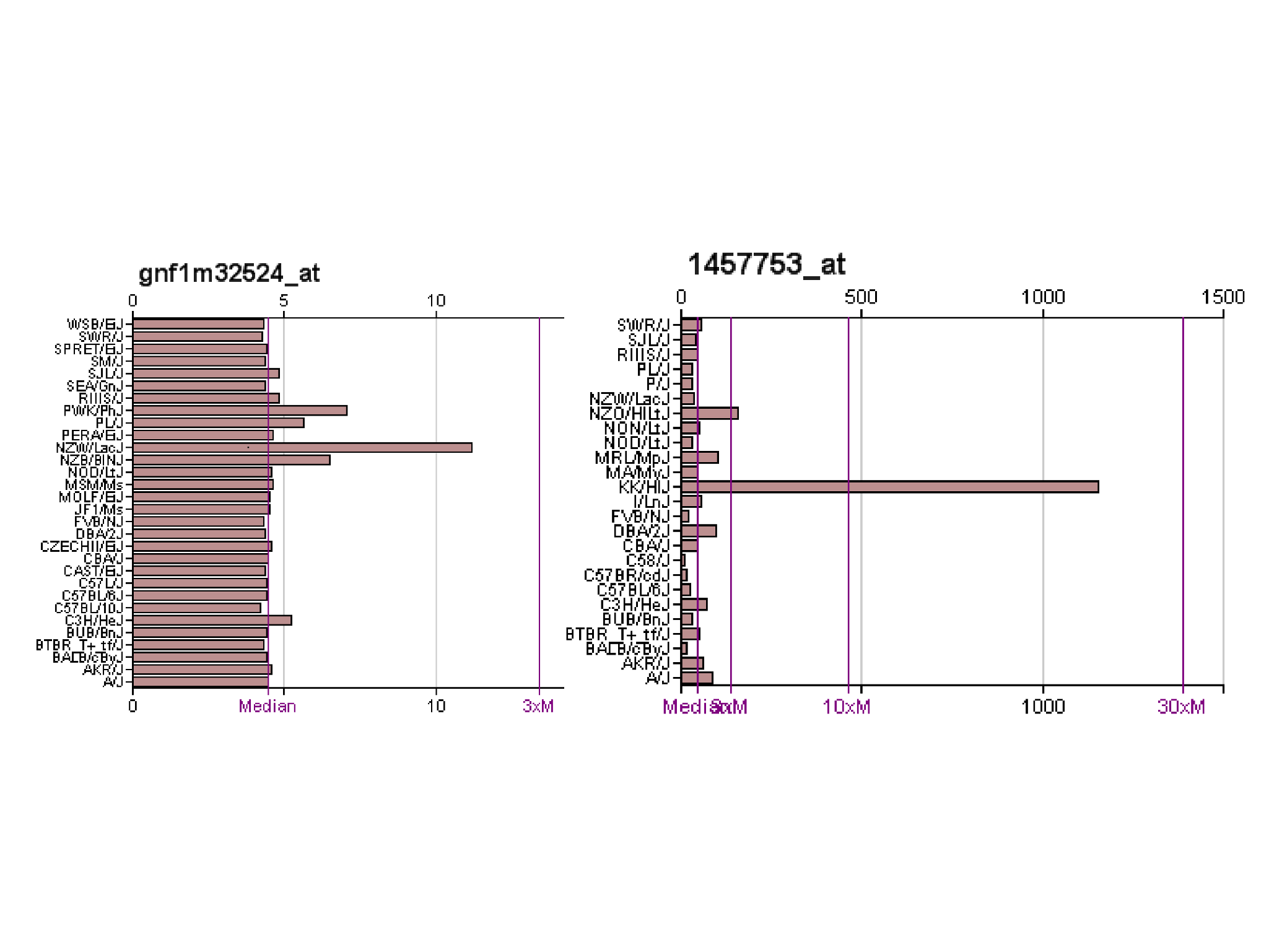

Supplement: Figure S1 — Toll-like receptor 13 (Tlr13) mRNA levels in adipose tissues from obese diabetic mouse strains. The bars show the relative expression of Tlr13 in the adipose tissues from multiple strains (http://biogps.gnf.org). Tlr3 expression patterns are shown from 2 eQTL analyses (gnf1m32524_at and 1457753_at) from the gene expression/acitivty chart of biogps from ‘Fat’ or ‘Adipose’. High Tlr13 expression is found in mouse strains that are also obese and diabetic (Supplemental Table S1 [70]). (TIFF) [file pone.0023944.s002.tiff]
